# The Importance and Manner of Sterilization of Dental Instruments

**Published:** 1899-03

**Authors:** L. A. Meyer

**Affiliations:** Oconomowoc, Wis.


ARTICLE VII.

### THE IMPORTANCE AND MANNER OF STERILIZATION OF DENTAL INSTRUMENTS.

L. A. MEYER, D. D. S., OCONOMOWOC, WIS.

Read before the Wisconsin State Dental Society.

1. The importance of sterilization of dental instruments to-day is recognized by all progressive dentists as a factor that we owe with a sense of justice and regard to our patrons.

It is our duty as dentists to treat our patients in such manner and with such instruments as we would have used were we looking for like services.

Being prompted with this spirit, and with the born necessity for better and more efficient aseptic and antiseptic services, by virtue of which we may triumph in the treatment of the diseased condition of the oral cavity, formerly unattainable.

There is no department of surgery in which the demand for aseptic procedure is more urgent than in dentistry, for the reason that all of our operations are performed upon septic or infected tissues.

We cannot extract a tooth, cleanse the canal of a pulpless tooth, excavate a cavity of decay or lance the gums, or touch any part of the oral cavity without our instrument becoming coated with a layer of infectious material. Therefore it is our duty to cleanse and sterilize our instruments to avoid the transmission of infectious matter from one patient to another.

The necessity of absolute cleanliness on the part of the dentist, of his hands, instruments, napkins, drinking glasses, rubber dam, in short everything which comes in contact with the patient's mouth is, as I have said, universally reeognized. And yet it is not at all difficult to find persons in the practice of dentistry who neglect this matter to an extent that is revolting to the taste, dangerous to the health, and certainly not creditable to the dental profession.

With regard to the transmission of disease by dental instruments, there have been so many cases reported in our dental and medical journals that the matter should be familiar to all of us. Such disturbances of the human body which have been traced to the action of mouth bacteria, and directly to the result of transmission by dental instruments are septicæmia, chronic pyæmia, meningitis, ostitis, syphilis, pyorrhœa, and many other disorders too numerous to mention. I can but refer you to a compiled list of cases by Dr. W. D. Miller, of Berlin, in "Cosmos," September number, 1891.

It is most fortunate that the gums in a healthy state offer so powerful a resistance to the invasion of pathogenic germs, although I regard it never safe to trust to the usually prcnounced immunity of the gums toward infections, since they under many abnormal conditions lose their power of resistance altogether.

And more than this, the mucous membrane of the mouth appears, under all conditions when slightly wounded, to furnish ready entrance to pathogenic germs.

We can never know what virus may be clinging to our instruments. Nor can we with certainty predict the result of a wound upon the gums, cheeks or lips with an unclean instrument.

It also stands to reason that in all operations within the oral cavity we should, as far as possible, sterilize the parts to be operated upon, since the danger of infection through the germs in the patient's own mouth is always present.

The demand for the adoption by the dentists of the same aseptic measures as observed by the general surgeon has constantly become more imperative.

And I sincerely hope that the performance of any bloody operation within the mouth, even to the extraction of a tooth, will be done with due regard to the principles of aseptic surgery.

Now, as members of a progressive profession, let me earnestly urge you to adopt such means of sterilization of your instruments used in daily practice, and to observe and practice the principles of asepsis in all operations within the oral cavity.

2. The question of sterilization of instruments is one which has given both surgeons and bacteriologists mucb to think and work upon, and only recently can it be said to have approached a definite solution.

Much credit is due to Dr. W. D. Miller, of Berlin, for work in this direction. And I wish to quote him as saying that boiling water will accomplish in two minutes as much as any chemical agent ordinarily used, in half an hour.

And in conclusion he says that "I regard an exposure of three minutes to boiling water sufficient for sterilizing smaller dental instruments, i. e., excavators, etc., and five minutes for forceps." Now there are many ways that one might adopt to accomplish a certain result. And each may have some special feature by which it merits recognition. After experimenting with several of the so-called dental and surgical instrument sterilizers, of which I have quite a collection at home, I came to the conclusion that the ideal dental instrument sterilizer would be found in some apparatus that made use of dry steam heat, or better still, some powerful gaseous compound.

As necessity is the mother of invention, I am pleased to be able to exhibit this invention, the Schering's formalin sterilizer, which has fulfilled my most sanguine expectation as an ideal for sterilization of dental instruments.

With this formalin sterilizer you have a method that is rapid, cheap, easy and sure of sterilization of instruments without in any way injuring them.

To verify the foregoing statement, can but state that I have conducted several experiments. By infecting instruments with various forms of micro-organisms, and after exposing them to the action of the gas to different intervals of time, and subjecting them to the usual proofs for being sterile or not, have come to this conclusion: That with one pastil or five grains of paraform, used in the Schering's formalin sterilizer, you can disinfect any infected dental instrument by an exposure of fifteen minutes to the formaldehyde gas generated from a five grain pastil.

Another advantage of these pastils is that it insures you with pure 100 per cent. formaldehyde gas in definite quantities.

Formaldehyde gas has proven to possess extraordinary power as a surface disinfectant, greater than that of any other known substance, This factor specially recommends it to us for use in sterilizing our instruments. It also has great penetrative powers in dry, loosely woven substances, as absorbent cotton, gauze, etc.

In the presence of moisture the penetrative power of this gas is materially lessened, and would say practically nil, This necessarily limits its action to surface disinfection.

Further, I claim this method to be an ideal one, as the lamp will burn in the closed chamber long enough to generate more than sufficient gas for its disinfection, and you use a definite amount of paraform and get a definite result in a given time.

Inexpensive, most convenient and absolutely certain in the destruction of all pathogenic organisms in the shortest time, and with a minimum of injury to the object of disinfection.

Of the several ways that formaldehyde gas and its solutions suggest themselves for use for the practical disinfection of instruments, I can but recommend and place reliance upon this method of evaporating paraform by heat in the manner suggested by using Schering's formalin lamp and sterilizer.

Solutions of formalin are not fit for practical use in disinfecting dental instruments, whereas, they may and do prove themselves efficient as disinfectants. The action of formalin and its solutions is such that it blackens and corrodes them. The precipitate you see in the bottom of bottles with test instruments in the various solutions of formalin is, I believe, hydrate or carbonate of iron. Furthermore, formalin is of acid reaction, and also coagulates albumen, even in attenuated solution. (Explains principle of disinfectant by Miller, Germany.) Have found lysol solution—two to four per cent. solution—efficient, and better than solution of formalin, as it will not corrode the instruments, it being an alkaline solution.

Now, I have mentioned formaldehyde, formalin and paraform several times, thinking that some of you may not have had access, or your attention called to the various literature upon the chemical and its practical use as an antiseptic and disinfectant. Wish to say that it was discovered by Von Hoffmann in 1867, and held as a chemical curiosity until 1888, when Loew discovered its antiseptic properties, and Berlioz and Trillot, in 1890, applied this property, and proved beyond doubt its practical use as an antiseptic and disinfectant.

Formaldehyde gas is prepared by subjecting methyl or wood alcohol to oxidation. Formalin is a forty per cent. of formaldehyde gas in water, and paraform is polermenized formaldehyde, and may be seen by evaporizing a solution of formalin, as a white precipitate on the bottom and the side of bottle. It is soluble in hot water, and further, by being heated again produces formaldehyde gas. Formaldehyde gas has the property of uniting with sulphureted nitrogenous products of decay, fermentation or decomposition, forming other chemical compounds which are antiseptic and sterile in their nature. To this property is due its great germicidal powers.

Formalin, forty per cent. solution of formeldehyde gas, coagulates albumen, hardens animal tissues without causing shrinkage or change in structure.

Since I have conducted several experiments with the formalin solution in the treatment of teeth, I wish to relate a few instances: Have used forty per cent. solution in treating a pulp tissue in accessible root-canals, placing a drop on pellet of cotton with solution in tooth from four to twenty-four hours, then filling, leaving pulp tissue in root undisturbed. In experiments on teeth out of the mouth treated in like manner, keeping them moist and in solution of formalin, found pulp tissue to be white and hard and unchanged with regard to shrinkage, am not prepared to say at this writing what the result would be, only judging from my experience in preservation of biological specimens, have reason to look for a favorable method of permanently fixing animal tissue thus treated from putrefaction five per cent. or ten per cent. solution better for penetrating hard tissue, as tooth substance. Have used a five per cent. solution of formalin in treating putrescent root canals and report but favorable results so far. One case gives rise to disturbances of an ædemic nature, but quieted down in three days, so I could fill tooth.—*Dental Review.*

